# Treatment Outcome of Tuberculosis Patients under Directly Observed Treatment Short Course and Factors Affecting Outcome in Southern Ethiopia: A Five-Year Retrospective Study

**DOI:** 10.1371/journal.pone.0150560

**Published:** 2016-02-26

**Authors:** Gebremedhin Gebrezgabiher, Gebremedhin Romha, Eyasu Ejeta, Getahun Asebe, Endalew Zemene, Gobena Ameni

**Affiliations:** 1 Jimma University, College of Health Sciences, P.O. Box 378, Jimma, Ethiopia; 2 Dilla University, College of Agriculture and Natural Resource, P.O. Box 419, Dilla, Ethiopia; 3 Samara University, College of Veterinary Medicine, P.O. Box 132, Samara, Ethiopia; 4 Wollega University, College of Health Sciences, P.O. Box 395, Nekemte, Ethiopia; 5 Gambella University, College of Agriculture and Natural Resource, P.O. Box 126, Gambella, Ethiopia; 6 Addis Ababa University, Aklilu Lemma Institute of Pathobiology, P.O. Box 1176, Addis Ababa, Ethiopia; The Foundation for Medical Research, INDIA

## Abstract

Tuberculosis (TB) is one of the major public health and socio-economic issues in the 21^st^ century globally. Assessment of TB treatment outcomes, and monitoring and evaluation of its risk factors in Directly Observed Treatment Short Course (DOTS) are among the major indicators of the performance of a national TB control program. Hence, this institution-based retrospective study was conducted to determine the treatment outcome of TB patients and investigate factors associated with unsuccessful outcome at Dilla University Referral Hospital, southern Ethiopia. Five years (2008 to 2013) TB record of TB clinic of the hospital was reviewed. A total 1537 registered TB patients with complete information were included. Of these, 942 (61.3%) were male, 1015 (66%) were from rural areas, 544 (35.4%) were smear positive pulmonary TB (PTB+), 816 (53.1%) were smear negative pulmonary TB (PTB-) and 177(11.5%) were extra pulmonary TB (EPTB) patients. Records of the 1537 TB patients showed that 181 (11.8%) were cured, 1129(73.5%) completed treatment, 171 (11.1%) defaulted, 52 (3.4%) died and 4 (0.3%) had treatment failure. The overall mean treatment success rate of the TB patients was 85.2%. The treatment success rate of the TB patients increased from 80.5% in September 2008-August 2009 to 84.8% in September 2012–May 2013. Tuberculosis type, age, residence and year of treatment were significantly associated with unsuccessful treatment outcome. The risk of unsuccessful outcome was significantly higher among TB patients from rural areas (AOR = 1.63, 95% CI: 1.21–2.20) compared to their urban counterparts. Unsuccessful treatment outcome was also observed in PTB- patients (AOR = 1.77, 95% CI: 1.26–2.50) and EPTB (AOR = 2.07, 95% CI: 1.28–3.37) compared to the PTB+ patients. In conclusion, it appears that DOTS have improved treatment success in the hospital during five years. Regular follow-up of patients with poor treatment outcome and provision of health information on TB treatment to patients from rural area is recommended.

## Introduction

Despite the availability of effective drugs, tuberculosis (TB) is still a global emergency and one of the major public health problems in the 21^st^ century [1]. It is not only a public health problem, but also a socio-economic issue [2]. According to the Global TB Report, in 2013 alone an estimated 9.0 million people developed TB and 1.5 million died from the disease, 360,000 of whom were HIV positive. Of the estimated 9 million people who developed TB in 2013, more than half (56%) were in the South-East Asia and Western Pacific Regions and 29% were in the African Region. The highest rates of cases and deaths out of the total population occurred in the African Region [3].

Ethiopia is among the 22 high TB burden and 27 high multi-drug resistant (MDR) TB burden countries in the world with an estimated TB incidence of 224 per 100, 000 populations in 2013. The prevalence and mortality of all forms of TB in Ethiopia were estimated to be 211 and 32 per 100,000 populations, respectively [3]. It remains a major public health problem claiming thousands of human lives every year.

The Directly Observed Treatment Short Course (DOTS) strategy, which allows patients to take their daily drugs under the observation of health professionals, thereby improving treatment compliance, has been known to increase TB cure rate [4]. In Ethiopia, a standardized TB prevention and control programme, incorporating DOTS, was started in 1992 as a pilot in Arsi and Bale zones of Oromia region. The DOTS strategy has been subsequently scaled up and implemented at national level [5]. Currently it is provided in almost all public hospitals and health centers as well as in private and non-governmental health facilities [6].

Treatment outcome is an important indicator of TB control programs [7], and monitoring and evaluation of treatment outcomes of TB patients is an integral part of the DOTS strategy [8]. In Ethiopia, previous studies conducted in Gondar areas [9, 10], Tigray [11], southern Ethiopia [12], Addis Ababa [13, 14] and Debre-Markos [15] evaluated treatment outcomes of TB. Socio-demographic factors including gender, age and residence of the patients and the form of TB have been reported to affect the treatment outcome and performance of DOTS services in these studies. Analysis of factors affecting treatment outcomes may help to improve performance of DOTS services and provide useful evidence for decision making in disease control programs [16]. Despite the provision of DOTS services in Dilla University Referral Hospital, the treatment outcome of TB patients and factors affecting treatment outcome have not been studied so far. Therefore, this study was initiated to determine the treatment outcome of TB patients and identify factors associated with unsuccessful outcome reviewing five years record of the patients in Dilla University Referral Hospital, southern Ethiopia.

## Methods

### Study Setting

A health facility-based study was conducted between November 2012 and May 2013 in Dilla University Referral Hospital, found in Dilla Town. Dilla Town is the administrative town of Gedeo Zone, located 359 km south of Addis Ababa. The hospital serves an estimated one million people in Gedeo Zone and its surroundings. It is a teaching hospital involved in training of medical and health science students besides provision of the health care services. Directly Observed Treatment Short Course TB clinic Unit is operates in the hospital under the National TB and Leprosy Control Program (NTLCP) of Ethiopia. In the hospital TB is diagnosed using routine sputum acid fast staining, radiological and histological examinations. No TB culture facilities were available during the time of data collection.

### Study design and data collection

A retrospective analysis of the profile and treatment outcome of all TB patients registered from September 1, 2008 to May 30, 2013 at DOTS TB Clinic was conducted. Information retrieved from the records includes socio-demographic profile of the patients, date of TB diagnosis and treatment outcome. Data were collected in data collection format prepared for this purpose. The data from the five-year records were retrieved by three of the research team members.

### Operational Definition

According to the standard definitions of the NLCP adopted from WHO [5], the following clinical case and treatment outcome operational terms were used:

#### Smear-positive pulmonary TB (PTB+)

A patient with at least two sputum specimens which were positive for acid fast bacilli (AFB) by microscopy, or a patient with only one sputum specimen which was positive for AFB by microscopy, and chest radiographic abnormalities consistent with active PTB.

#### Smear-negative pulmonary TB (PTB-)

A patient with symptoms suggestive of TB, with at least two sputum specimens which were negative for AFB by microscopy, and with chest radiographic abnormalities consistent with active PTB, or a patient with two sets of at least two sputum specimens taken at least two weeks apart, and which were negative for AFB by microscopy, and radiographic abnormalities consistent with PTB and lack of clinical response to one week of broad spectrum antibiotic therapy.

#### Extra pulmonary TB (EPTB)

This included TB of organs other than the lungs, such as lymph nodes, abdomen, genitourinary tract, skin, joints and bones, the meninges and others. Diagnosis of EPTB was based on fine needle aspiration cytology or biochemical analyses of cerebrospinal/pleural/ascitic fluid or histopathological examination or strong clinical evidence consistent with active EPTB, followed by a decision of a clinician to treat with a full course of anti-TB chemotherapy. In all the cases of EPTB, sputum examinations and chest radiographs were used to rule out involvement of the lung parenchyma. This hospital lacks the facilities for culture and drug susceptibility testing.

According to WHO, treatment outcomes were categorized into:

1. *Successful outcome*: If TB patients were cured (negative smear microscopy at the end of treatment and on at least one previous follow-up test) or completed treatment with resolution of symptoms.
2. *Unsuccessful outcome*: If treatment resulted in treatment failure (remaining smear-positive after 5 months of treatment), defaulted (patients who interrupted their treatment for two consecutive months or more after registration), or died.

### Inclusion and exclusion criteria

All forms of TB cases which were registered in the TB clinic of the hospital were included in the study. However, registries in which treatment outcomes were missing, and patients who transferred to other districts were excluded from the treatment outcome evaluation, as information on their treatment outcome was not available.

### Statistical analysis

Data were entered, cleaned and analyzed using SPSS for windows, version 20. To ensure quality of the data, two individuals independently cross-checked each entry. Proportions with 95% confidence intervals, Odds ratio and Chi-square tests were employed to compare different groups for categorical data. Multivariable logistic regression model was used to analyze the association between the outcome variable and potential predictor variables. P-value <0.05 were considered statistically significant.

### Ethical statement

The study was ethically approved from the Institutional Review Board of Aklilu Lemma Institute of Pathobiology, Addis Ababa University (Reference No IRB/01/2012-13). Permission was sought from the hospital administration before data collection. Patient records/information was anonymized and de-identified prior to analysis to ensure confidentiality of individual patient information.

## Results

### Socio-demographic and clinical characteristics of the patients

**Fig 1:**
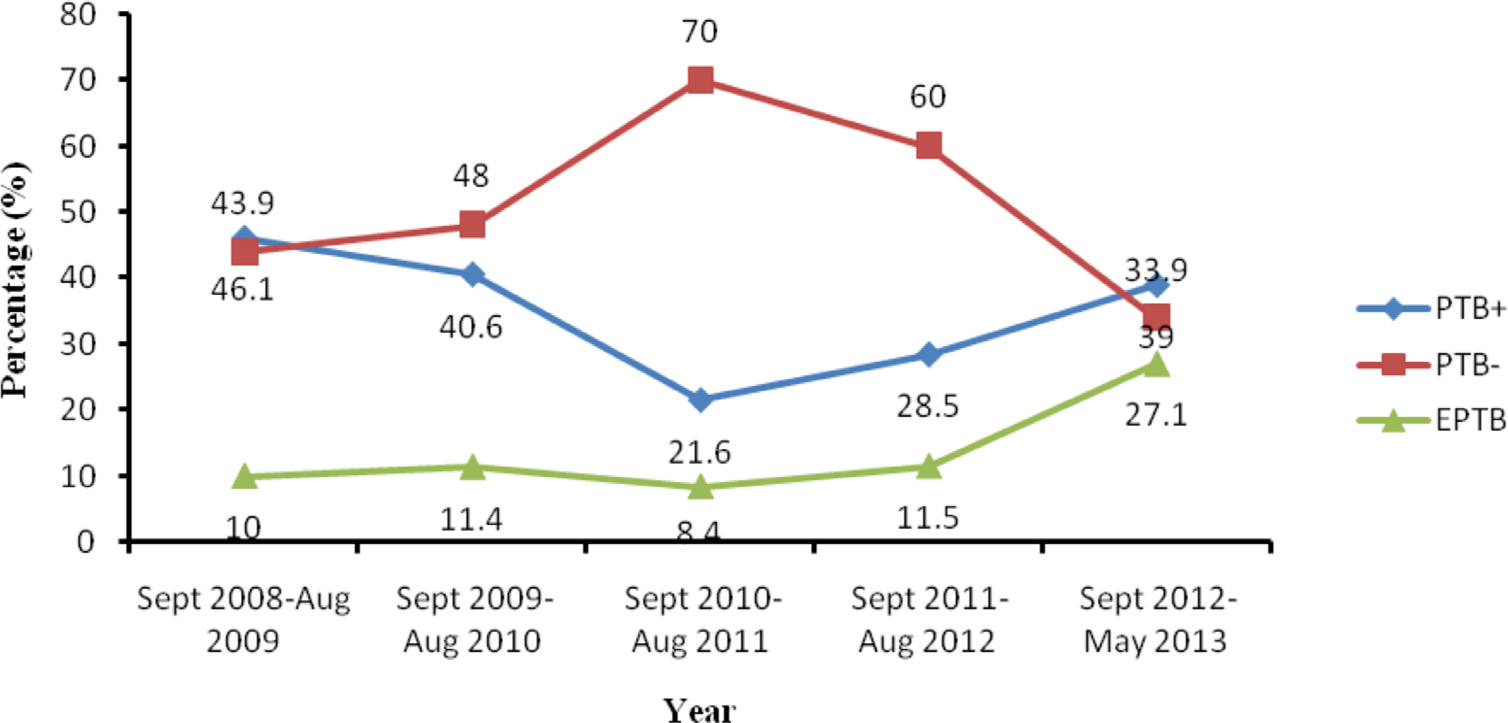
Trend of all types of registered TB cases (n = 1537) in Dilla University Referral Hospital, Southern Ethiopia, 2008–2013. PTB+, Smear positive pulmonary tuberculosis; PTB-, smear negative pulmonary tuberculosis; EPTB, Extra pulmonary tuberculosis; Sep, September; Aug, August.

Socio-demographic profile and other clinical information of a total of 1537 registered TB patients was obtained and included for further analysis (Table 1). Majority of the patients were male (61.3%) and rural residents (66%). Two hundred and eight(13.5%), 456(29.7%), 416(27.1%), 220(14.3%), 127(8.3%), 60(3.9%) and 50(3.3%) of the patients were within the age group 0–14, 15–24, 25–34, 35–44, 45–54, 55–64 and above 65 years, respectively. Clinical record of the patients showed that more than half (53.1%), 544(35.4%) and 177(11.5%) were PTB-, PTB+ and EPTB patients, respectively. The five-year trend of all forms of TB cases is shown in Fig 1.

**Table 1:** Socio-demographic characteristics of the registered TB cases (n = 1537) in Dilla University Referral Hospital, Southern Ethiopia, 2008–2013.

TB type	Sex		Age (years)							Residence		Total (%)
	M ale	Female	≤14	15–24	25–34	35–44	45–54	55–64	≥65	Urban	Rural	
**PTB+**	325(34.5)	219(36.8)	64(30.8)	180(39.5)	147(35.3)	73(33.2)	47(37.0)	16(26.7)	17(34.0)	191(36.6)	353(34.8)	544(35.4)
**PTB-**	507(53.8)	309(51.9)	120(57.7)	213(46.7)	209(50.2)	129(58.6)	77(60.6)	39(65.0)	29(58.0)	280(53.6)	536(52.8)	816(53.1)
**ETB**	110(11.7)	67(11.3)	24(11.5)	63(13.8)	60(14.4)	18(8.2)	3(2.4)	5(8.3)	4(8.0)	51(9.8)	126(12.4)	177(11.5)
**All TB cases**	942(61.3)	595(38.7)	208(13.5)	456(29.7)	416(27.1)	220(14.3)	127(8.3)	60(3.9)	50(3.3)	522(34.0)	1015(66.0)	1537(100)

PTB+, Smear positive pulmonary tuberculosis; PTB-, smear negative pulmonary tuberculosis; EPTB, Extra pulmonary tuberculosis

### Treatment outcome of TB and its trend

A total of 1537 TB patients’ data recorded from September 1, 2008 to May 30 2013 were analyzed. Out of the total TB patients, 11.8%, 73.5%,11.1%,3.4%, and 0.3% were cured, completed treatment, defaulted, died and with treatment failure, respectively. The cure rate of TB patients steadily increased from 1.6% in September 2008–August 2009, to 9.6% in September 2009–August 2010, to 14% in September 2010–August 2011, to 20% in September 2011–August 2012 and to 31.4% in September 2012– May 2013. Overall, improving trend of successful TB treatment outcome was observed during the five years. Yearly treatment outcome of the TB patients is shown in Table 2.

**Table 2:** Trend of treatment outcome of all forms registered TB cases (n = 1537) in Dilla University Referral Hospital, Southern Ethiopia, 2008–2013.

Treatment outcome	Year					Total
	Sept 2008-Aug 2009	Sept 2009- Aug 2010	Sept 2010- Aug 2011	Sept 2011- Aug 2012	Sept 2012-May 2013	
**Cured**	6(1.6)	43(9.6)	55(14.0)	40(20.0)	37(31.4)	181(11.8)
**Completed**	300(78.9)	340(76.2)	292(74.3)	134(67.0)	63(53.4)	1129(73.4)
**Total**	**306 (80.5)**	**383(85.8)**	**347(88.3)**	**174(87)**	**100(84.8)**	**1310(85.2)**
**Defaulted**	64(16.8)	51(11.4)	33(8.4)	16(8.0)	7(5.9)	171(11.1)
**Death**	10(2.6)	10(2.2)	13(3.3)	9(4.5)	10(8.5)	52(3.4)
**Failure**	0(0.0)	2(0.4)	0(0.0)	1(0.5)	1(0.8)	4(0.3)
**Total**	**74(19.4)**	**63 (13)**	**46(11.7)**	**26(13)**	**18(13.6)**	**227(14.8)**

Sep, September; Aug, August

The default rate declined during the five years from 16.8% in September 2008 –August 2009 to 5.9% in September 2012–May 2013. However, the trend of death rate showed an increase from 2.6% from September 2008 –August 2009 to 8.5% in September 2012–May 2013 as shown in Table 2.

### Treatment success rate and its associated predictors

The overall mean treatment success of the TB patients (n = 1537) was 85.2% across the five years. The trend of treatment success rate of all forms TB patients showed an increase from 80.5% in September 2008–August 2009 to 84.8% in September 2012– May 2013 as depicted in Table 2. The treatment success rate was similar in males (84%) and females (86%). The treatment success rates were 89.2%, 83.3% and 81.9% among PTB+, PTB- and EPTB patients, respectively. The association of socio-demographic risk factors on treatment outcomes is presented in Table 3.

**Table 3:** Socio-demographic factors and treatment outcome among TB patients treated in Dilla University Referral Hospital, Southern Ethiopia, 2008–2013.

Characteristics		Total number (%) of TB cases	Number (%) with unsuccessful outcome	χ2-value	P-value
**Sex**				0.96	0.328
	Female	595(38.7)	95(16.0)		
	Male	942(61.3)	132(14.0)		
**Residence**				10.56	0.001
	Urban	1015(66)	128(12.6)		
	Rural	522(34)	99(19.0)		
**TB type**				10.53	0.005
	PTB+	544(35.4)	59(10.8)		
	PTB-	816(53.1)	136(16.7)		
	EPTB	177(11.5)	32(18.1)		
**Age (Years)**				29.98	<0.001
	≤14	208(13.5)	35(16.8)		
	15–24	456(29.7)	53(11.6)		
	25–34	416(27.1)	52(12.5)		
	35–44	220(14.3)	43(19.5)		
	45–54	127(8.3)	16(12.6)		
	55–64	60(3.9)	21(35.0)		
	≥65	50(3.3)	7(14.0)		
**Year**				10.28	0.036
	Sept 2008-Aug 2009	380(24.7)	74(19.5)		
	Sept 2009- Aug 2010	446(29)	63(14.1)		
	Sept 2010- Aug 2011	393(25.6)	46(11.7)		
	Sept 2011- Aug 2012	200(13)	26(13.0)		
	Sept 2012-May 2013	118(7.7)	18(15.3)		

Sep, September; Aug, August

Multivariable logistic regression revealed that after adjusting for other variables (Table 4), the risk of unsuccessful TB treatment outcome was significantly higher among TB patients from rural areas (AOR = 1.63, 95% CI: 1.21–2.20) compared to their urban counterparts. Moreover, poor treatment outcome was observed in PTB- patients (AOR = 1.77, 95% CI: 1.26–2.50) and EPTB (AOR = 2.07, 95% CI: 1.28–3.37) compared to the PTB+ patients.

**Table 4:** Logistic regression analysis of predictor variables of treatment outcome among TB patients in Dilla University Referral Hospital, Southern Ethiopia, 2008–2013.

Characteristics		Total number (%) of TB cases	Number (%) with unsuccessful outcome	AOR (95% CI)	P-value
**Sex**					
	Female	595(38.7)	95(16.0)	1.00 (Reference)	
	Male	942(61.3)	132(14.0)	0.90 (0.67–1.21)	0.496
**Residence**					
	Urban	1015(66)	128(12.6)	1.00(Reference)	
	Rural	522(34)	99(19.0)	**1.63 (1.21–2.20)**	0.001
**TB type**					
	PTB+	544(35.4)	59(10.8)	1.00(Reference)	
	PTB-	816(53.1)	136(16.7)	**1.77 (1.26–2.50)**	0.001
	EPTB	177(11.5)	32(18.1)	**2.07 (1.28–3.37)**	0.003
**Age group (year**)					
	≤14	208(13.5)	35(16.8)	1.00(Reference)	
	15–24	456(29.7)	53(11.6)	0.73 (0.46–1.18)	0.197
	25–34	416(27.1)	52(12.5)	0.78 (0.48–1.25)	0.300
	35–44	220(14.3)	43(19.5)	1.34 (0.80–2.20)	0.269
	45–54	127(8.3)	16(12.6)	0.78 (0.41–1.50)	0.461
	55–64	60(3.9)	21(35.0)	**3.06 (1.58–5.92)**	0.001
	≥65	50(3.3)	7(14.0)	0.94 (0.39–2.29)	0.890
**Year**					
	Sept 2008-Aug 2009	380(24.7)	74(19.5)	1.00(Reference)	
	Sept 2009- Aug 2010	446(29)	63(14.1)	**0.64 (0.44–0.94)**	0.022
	Sept 2010- Aug 2011	393(25.6)	46(11.7)	**0.48 (0.32–0.73)**	0.001
	Sept 2011- Aug 2012	200(13)	26(13.0)	**0.57 (0.35–0.94)**	0.029
	Sept 2012-May 2013	118(7.7)	18(15.3)	0.70 (0.39–1.26)	0.232

OR, Odds Ratio; AOR, Adjusted Odds ratio; CI, Confidence interval; Sep, September; Aug, August

## Discussion

In this retrospective study, complete information was extracted from TB registration documents for a total of 1537 registered TB patients. Most of the patients were male in contrast to previous studies done in Gondar [9] and Gambella [17]. This could be due to underutilization of the DOTS service by females or higher proportion of males being exposed to the infection in the area. A study conducted in Bangladesh on access to TB diagnosis and treatment also documented that women have poorer access to public outpatient clinics than men [18].

TB-associated morbidity and mortality occurs mainly in the economically productive age group [19]. In this study, 79.4% of the registered TB patients fall in the age range between 15–55 years, the most productive age group. This may pose challenges to the social and economic development of the community in the area and the nation at large.

Our study showed that an 11.8% and 73.5% of the TB patients attending DOTS were cured and completed treatment, respectively. These account an overall treatment success rate of 85.2%, which was similar with the 2013 international treatment success rate of 86% among all new TB cases [3] and studies done elsewhere [20,21]. The treatment success rate obtained was slightly lower than those reported from Dabat (87.8%) [10] and Tigray (89.2%) [11] in northern Ethiopia. However, the treatment success rate was lower compared to the WHO treatment success report for Ethiopia (91%) [3] and the national TB success rate (91.4%) [22]. On the other hand, the treatment success rate obtained in this study was higher than reports from several areas in Ethiopia, in which treatment success rates ranging from 26% to 80.7% were reported [9, 13–15, 17, 23, 24, 25]. The comparably more successful treatment outcome recorded in this study shows the promising performance of institutional DOTS in TB control program in the area. The increasing trend of TB treatment success from 2008 to 2013 obtained in this study was similar with a study in done in Addis Ababa [13]. This might be due to improved adherence of TB patients to treatment that may signify the importance of the DOTS strategy.

This study also revealed default, death and treatment failure rate of 11.1%, 3.4% and 0.3%, respectively. These constituted an overall unsuccessful TB treatment outcome rate of 14.8%, which was higher than the 10.8% unsuccessful treatment outcome reported from Tigray region [11]. Comparably higher (16.7%) unsuccessful outcome has been reported from southern Ethiopia [12]. This could be due to difference in duration of study period, sample size and study settings. The default rate in this study comprised major portion of the unsuccessful outcome in TB treatment. A similar [26] and higher [27] default rates were reported elsewhere. However, the default rate in this study was higher than the default rates reported from China (5.9%) [25], Sweden (7%) [28] and Malawi (6.6%) [29].The death rate among the TB patients in this study was 3.4%, which is lower than death rates (6% to25%) previously reported from other regions of the world[24, 26, 27, 29, 30]. A lower death rate (2.8%)has been report from Guangzhou, China [25]. The lower treatment failure rate in this study might be due to good treatment adherence and low prevalence of MDR-TB. Similar magnitude of treatment failure (0.2%) has been reported from Gondar [9].

Consistent with a previous study [23], patients from rural areas attending DOTS in the health facility had significantly poorer treatment outcome compared to patients from urban areas. It has been suggested that patients from rural areas may have lower awareness of TB treatment and the long distance between their homes and the treatment centers could contribute to lower treatment success [31]. However, a contrary report has recently been documented in Sidama Zone [16].

This study showed that PTB- and EPTB patients had significantly lower treatment success rate compared to the PTB+ patients. This could be due high rate of HIV co-infection in these group of patients [15, 32, 33], which may decrease the treatment success and increase mortality. This study also showed that the patients in the age group of 55–64 years had significantly low TB treatment outcome. This might be associated with increased co-infections with other diseases that might contribute to poorer treatment outcome.

The findings of this study should be seen in light of the fact that the study incorporated data of patients with completed information of their treatment outcome at the hospital. Tuberculosis patients transferred to other health facilities were not included. Moreover, important patient information which could affect TB treatment outcome, including HIV sero-status and co-morbidity with other chronic illnesses, distance from the treatment center, occupation and educational level of the patients were not obtained, thus not included in the analysis. Hence, these limitations need to be considered while interpreting the findings.

## Conclusion

The DOTS strategy appears to have improved TB treatment success in the hospital across five years. The mean treatment success rate of all registered patients was satisfactory and in line with the WHO target. The unsuccessful treatment outcome was significantly associated with residence, age and type of TB and year of treatment. Regular follow of patients with unsuccessful outcome and awareness creation through health education for rural patients in the course of treatment is vital. Moreover, attention should be given to regular sputum follow up tests for registered PTB+ cases and proper registration of record the results of treatment outcome.
